# Comparing Point-of-Care Ultrasound in Multiple Body Positions in Dogs to Key Cardiac Measurements by Echocardiography

**DOI:** 10.3390/vetsci13040367

**Published:** 2026-04-09

**Authors:** Ida M. Kornevi, Allison K. Masters, Aaron Rendahl, Rosalind S. Chow

**Affiliations:** Department of Veterinary Clinical Sciences, University of Minnesota, 1365 Gortner Avenue, St Paul, MN 55108, USA; maste289@umn.edu (A.K.M.); rend0020@umn.edu (A.R.); rchow@umn.edu (R.S.C.)

**Keywords:** canine, echocardiogram, recumbency, point-of-care ultrasound

## Abstract

Cardiac point-of-care ultrasound (cPOCUS) is used to obtain key information about the heart’s structure and function when an echocardiogram is not available. This prospective, cross-sectional study aimed to compare fractional shortening (FS%) and left-atrium-to-aorta ratio (LA:Ao) obtained by cPOCUS in different body positions to echocardiography. Thirty-nine dogs had cPOCUS performed in three different body positions: left lateral recumbency (RT), right lateral recumbency (LT), and standing or sternal recumbency (RST). The cPOCUS values from each body position for FS% and LA:Ao were tested for agreement with the echocardiogram by Bland-Altman plots, correct clinical assessment by generalized estimated equation models, and quality score of the cPOCUS images as a percentage. Bland-Altman analysis showed a positive bias for FS% (0.9% to 9.8%) and both positive and negative bias for LA:Ao (within 0.2) for the cPOCUS values. The correct clinical assessment was made in the majority of cases for FS% in RST (67%) and RT (67%), and for LA:Ao in RST (55%). The clinical assessment was more often correct when the image quality score was higher. Intraclass correlation showed good agreement (≥0.61) between investigators for FS% in all body positions and LA:Ao in RT and RST. This study showed that cPOCUS performed from the right hemithorax can provide estimates and correct clinical assessment of FS% and LA:Ao. Obtaining measurements in the LT position is not recommended.

## Simple Summary

Cardiac point-of-care ultrasound (cPOCUS) is a useful diagnostic tool in dogs that helps veterinarians obtain key information about the heart’s structure and function. Although a complete ultrasound of the heart (an echocardiogram) by a board-certified cardiologist is the best method of diagnosing heart disease in dogs, this is not always possible. Echocardiography in dogs is traditionally performed with the dog lying on their side with the ultrasound probe placed on the downward side of the dog and aiming upward toward the heart. In contrast, cPOCUS is commonly performed in many different body positions. Other ways of imaging the heart have shown changes in measurements of the heart, dependent on the position of the patient. It is unknown whether the measurements obtained in different body positions are comparable to those obtained through echocardiography. In this study, we show that cPOCUS performed in dogs that are either standing or lying on their left side with the ultrasound probe placed on the right side of their chest is better than the dog lying on the right side with the ultrasound probe on the left. The views are not fully interchangeable. cPOCUS, when performed from the right side of the chest, can be used to determine whether the heart appears normal or abnormal to help in the diagnosis of dogs with suspected cardiac disease.

## 1. Introduction

Point-of-care ultrasound is commonly utilized during patient assessment in veterinary medicine to aid in the differentiation between a wide array of diseases in acutely presenting patients and to guide emergency treatment. As a cage-side diagnostic tool, it is a convenient and rapid alternative to many other types of imaging [1,2]. Cardiac point-of-care ultrasound (cPOCUS) [3] describes the visualization of a limited number of sonographic views to provide specific information about cardiac structure and function [1]. It is commonly performed by non-cardiology specialists or general practitioners using a micro-convex probe and a general, portable ultrasound machine to facilitate assessment of other structures. Its main benefits include a rapid assessment for pericardial effusion and structural heart disease, leading to left- and/or right-sided congestive heart failure [2]. Having the ability to perform a quick, informative assessment is particularly beneficial in patients presenting for respiratory distress, collapse, or shock, where there is a need to distinguish between cardiac and non-cardiac etiologies.

When it comes to evaluating the structure and function of the heart, an echocardiogram performed by a board-certified cardiologist is the gold standard. To obtain optimal visualization of the heart chambers during an echocardiogram, the patient should lie on their left or right side on a cut-out table, and the probe should approach from underneath [4]. cPOCUS can be used as a screening tool when echocardiography is not an option because of patient or practical reasons. Since unstable patients may not tolerate restraint, cPOCUS is commonly performed in sternal, standing, and lateral recumbency positions [1,2,5,6]. However, it is unclear whether the lack of positional standardization introduces variability that affects the measurements and the resulting conclusions.

Radiography and CT studies in cats and dogs have reported small differences in the positioning and subsequent measurements of the heart, including vertebral heart score, depending on patient recumbency [7,8,9,10]. Since echocardiography relies upon proper orientation of the probe relative to the heart to achieve specific sonographic views, this raises the question of whether the key cardiac measurements obtained during cPOCUS are altered by changes in body position, which would result in discrepancies when they are compared to echocardiography. Recumbency has not been shown to affect the left-atrial-to-aorta ratio (LA:Ao) or left atrial diameter in cats undergoing cPOCUS in lateral versus sternal recumbency [6]. However, whether there are measurable differences in these cPOCUS variables in dogs in different body positions has not previously been reported to the authors’ knowledge.

Apart from the unknown potential effect of patient positioning on cardiac measurements in dogs, different recumbencies may also affect image quality by altering the acoustic windows through which the heart may be optimally visualized. Positioning the ultrasound probe by the cardiac notch, formed in the space between the right cranial and middle lung lobes, and at the level of a palpable apex beat, is the most common approach in dogs [1,2,11]. Ultrasound waves are predominantly reflected when they encounter air, for example, at the pleural pulmonary boundary in the inflated lung, resulting in a hyperechoic line with no further tissue penetration [1]. Placing the probe at the cardiac notch is therefore thought to minimize the interference of air artefact when imaging from the right hemithorax. Imaging of the heart from the opposite side can be performed at the ventral-most part of the left hemithorax, but this space is smaller than the right pericardial window [11], and superimposition of the lungs leads to poor image acquisition. As lung volume and aeration change with body position [7], so may the size of the acoustic windows, and whether the quality of images obtained during cPOCUS varies with patient recumbency remains unreported.

Two key calculations that are frequently performed based on measurements obtained during cPOCUS are fractional shortening (FS%) and LA:Ao, which provide clinically important information about cardiac structure and function [1,2]. The FS% is a two-dimensional quantification of left ventricular systolic function calculated from measurements of the left ventricular internal diameter at end-diastole (LVIDd) and end-systole (LVIDs). The LA:Ao ratio indexes left atrial size to the patient’s aorta. Assessment of LA:Ao is included in the ACVIM guidelines for classifying myxomatous mitral valve disease [12], and it has been shown to allow differentiation between pulmonary edema secondary to non-cardiac or cardiac causes [13,14,15]. The LA:Ao is calculated by measuring the aortic dimension in short axis at the level of the aortic valve and dividing the value by the left atrial dimension, in early diastole. Both FS% and LA:Ao could aid veterinarians assessing and treating dogs presenting with clinical signs potentially attributable to cardiac disease by providing focused but helpful diagnostic information that would not be possible without a full echocardiogram. While these two variables are traditionally obtained via the right parasternal short axis view (RPSAX) with patients lying in right lateral recumbency, these cardiac structures may also be visualized from other approaches and body positions.

In this study, the main goal was to compare the agreement of FS% and LA:Ao in dogs between images obtained via the RPSAX view during echocardiography to those acquired by cPOCUS in three different positions: (a) the RPSAX view in dogs lying in left lateral recumbency (RT), (b) the RPSAX view in dogs in sternal position (RST), and (c) from the left hemithorax with dogs lying in right lateral recumbency (LT). As supplementary goals, we wanted to see if cPOCUS assessments of FS% and LA:Ao from various body positions would yield the same clinical assessment as echocardiography, and whether it would affect the imaging quality. We hypothesized that FS% and LA:Ao measured with cPOCUS from different body positions would be similar to those acquired by echocardiography, and that the cPOCUS measurements would be able to accurately distinguish between normal and abnormal results in the majority of cases, independent of body position. We also hypothesized that image quality scores would be lower in images obtained from the left hemithorax.

## 2. Materials and Methods

This prospective, blinded, cross-sectional study received ethical approval from the Institutional Animal Care and Use Committee (protocol ID 2310-41487A). Dogs referred to the cardiology service at the University of Minnesota Veterinary Medical Center for an echocardiographic assessment for a suspected cardiovascular abnormality or screening for breeding purposes were enrolled on a voluntary basis. Power analysis was performed prior to enrollment by precision-based sample size calculation for a proportion of agreement. The results showed that, with a sample size of 30, we would have 80% power to estimate the proportion of agreement to within 16 percentage points of the true value, using a 95% confidence interval and assuming the true proportion is 80% or higher. Enrollment occurred on selected days when the primary enrolling investigator (IK) was available to participate in recruitment. As the recruitment target of 30 dogs was met much earlier than anticipated, recruitment continued until the entire period for data collection was completed (February through June, 2024).

Dogs over one year of age, of any breed, sex, and neuter status, that presented to the cardiology service for evaluation for known or possible heart disease, were eligible for enrollment. Informed consent was obtained from the owner prior to the echocardiogram. Dogs that were younger than one year of age or intolerant of echocardiograms were excluded. There was no exclusion based on underlying heart disease or stage of disease, as long as the dog tolerated restraint well. The decision to use sedation prior to the echocardiogram, as well as drug selection, was at the discretion of the cardiology clinician overseeing the case. Patient demographics, weight, and echocardiographic diagnosis were recorded. The echocardiogram was performed by either an ACVIM-boarded cardiologist or a supervised cardiology resident. After the echocardiogram, cPOCUS was performed by one of the authors (IK), deemed proficient in acquiring the two evaluated views after training with an ACVIM board-certified cardiologist (AM). No measurements or diagnoses were made between the echocardiogram and the acquisition of the cPOCUS images. All images were collected with a cardiovascular ultrasound machine (GE Vivid E95) and stored in a cardiac ultrasound software program (GE EchoPAC PC SW Version 204). Three different phased-array probes were used (12s-RS, 7s-RS, 5MS-D matrix), and the probe utilized was at the discretion of the cardiology clinician based on patient size. Both isopropyl alcohol and acoustic coupling gel were used at the discretion of the clinician. No dogs in the study were shaved for their echocardiogram or cPOCUS as per standard protocol at the study institution.

**Figure 1:**
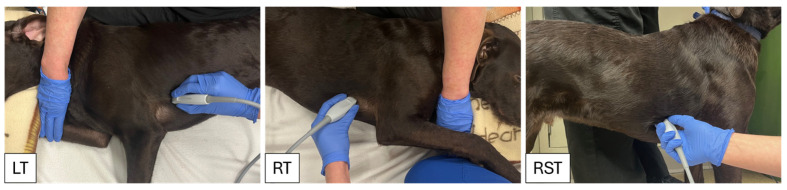
Depiction of the three different body positions and probe placements used in the study. LT—patient in right lateral recumbency with the ultrasound probe on the left hemithorax; RT—patient in left lateral recumbency with the ultrasound probe on the right hemithorax; RST—patient is standing or in sternal recumbency with the ultrasound probe on the right hemithorax.

The cPOCUS views were obtained in three different positions for each dog: (a) placing the probe on the right hemithorax (RT) while the patient lay in left lateral recumbency, (b) placing the probe on the right hemithorax while the patient stood or lay in sternal recumbency (RST), depending on patient preference, and (c) placing the probe on the left hemithorax (LT) while the patient lay in right lateral recumbency. The different body positions are depicted in Figure 1. The recumbency order for obtaining cPOCUS images was not standardized and was based on patient-specific factors. For each body position, two short-axis cardiac views were obtained, with the first at the level of the left ventricular papillary muscles for FS% measurements and the second at the level of the aortic valve for LA:Ao measurements. In RT and RST, FS% and LA:Ao views were both obtained from the RPSAX at the level of the left ventricular papillary muscles and the aortic valve, respectively. In LT, FS% was obtained from the left parasternal view with the ventricles in short-axis at the level of the left ventricular papillary muscles. LA:Ao was obtained by starting at the left parasternal five-chamber view at the level of the aortic valve and continuing to fan the probe until visualization of the left atrium and aorta was optimized. While LA:Ao measurements are not performed routinely during comprehensive echocardiographic studies from this view, patient comorbidities in the emergency setting can preclude right thoracic imaging; therefore, this view was included in the study. All cPOCUS images were performed in 2-D mode and recorded as cine loops. The start of each cine loop was determined by the investigator acquiring the cPOCUS images and automatically stopped after three heartbeats based on simultaneous lead II ECG. Three cine loops were recorded for each of the two views (FS% and LA:Ao) and three body positions (LT, RT, RST).

All measurements used in this study, for both cPOCUS and the echocardiogram, were performed off-cart in the cardiac ultrasound software program in 2-D mode. The echocardiogram images were measured on the same day the images were captured by the ACVIM-boarded cardiologist or supervised cardiology resident who obtained them. The cPOCUS images were independently measured by the three investigators (IK, AM, RC) after all dogs had been enrolled. The investigators consisted of one emergency and critical care resident, one board-certified criticalist, and one board-certified cardiologist. Each investigator was blinded to all patient information, including demographics, diagnoses, and body position, by having another person retrieve the images in the software program, shielding the patient information during measurements, and not revealing any prior echocardiographic measurements, assessments, or diagnoses. All measurements were obtained using the inner edge-to-inner-edge method. LVIDd was defined as the maximum chamber dimension at or near the onset of the QRS complex. LVIDs was defined as the minimum chamber dimension [16]. The formula used to then calculate FS% was (LVIDd − LVIDs)/LVIDs × 100, and the normal value was defined as between 25–45% [17]. The aortic diameter (Ao) and left atrium diameter (LA) for the calculation of LA:Ao were obtained using the Scandinavian method [18]. If visualized, the measurements were performed at the earliest point when the aortic valves closed. If unable to visualize the aortic valves, the measurements were done in early diastole—defined as after the end of the T wave and before the mid-way point between the T wave and P wave [16]. The formula used to then calculate LA:Ao was LA/Ao, and the normal value was defined as ≤1.6 [16].

If the measurement conditions could not be met as indicated, no measurements were performed. Measurements during dysrhythmias were avoided when possible, including but not limited to ectopic beats, ventricular premature complexes, and atrioventricular (AV) block. For dogs with pacemakers, measurements during paced beats were included. If the measurer was unable to obtain the paired measurements required to calculate FS% or LA:Ao, for example, if the measurer measured LIVDs but not LVIDd, then neither measurement was recorded.

**Figure A1:**
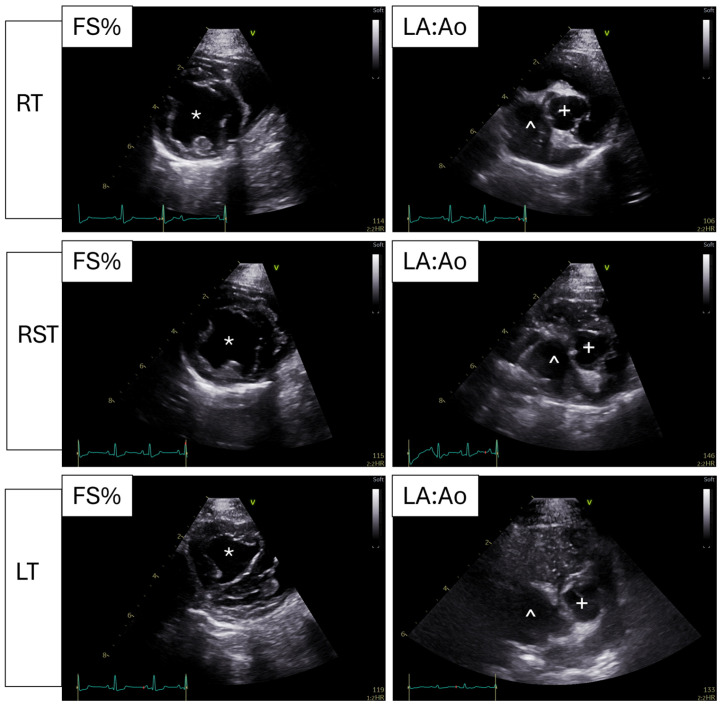
Images obtained by cardiac point-of-care ultrasound (cPOCUS) for measurements of fractional shortening (FS%) and left-atrium-to-aorta ratio (LA:Ao) in different body positions. *—left ventricle; ^—left atrium; +—aorta, v—ultrasound orientation marker; LT—patient in right lateral recumbency with the ultrasound probe on the left hemithorax; RT—patient in left lateral recumbency with the ultrasound probe on the right hemithorax; RST—patient is standing or in sternal recumbency with the ultrasound probe on the right hemithorax.

For each of the three body positions, three cine loops were recorded for the calculation of FS%, and three cine loops were recorded for the calculation of LA:Ao. For each cine loop, measurements were obtained from three consecutive heartbeats when possible, or two consecutive heartbeats if the relevant portion of the third waveform was not fully captured on ECG. All LVIDd and LVIDs, and LA and Ao measurement pairs that were able to be obtained were recorded, along with the heart rate and rhythm of each cine loop. The FS% or LA:Ao were calculated separately for each heartbeat and then averaged within each cine loop to get one value per cine loop for each view. Each dog could therefore have up to three FS% values and up to three LA:Ao values in each body position per investigator. This process was then repeated for the next two body positions. Representative cPOCUS images in each body position can be found in Figure A1.

**Figure 2:**
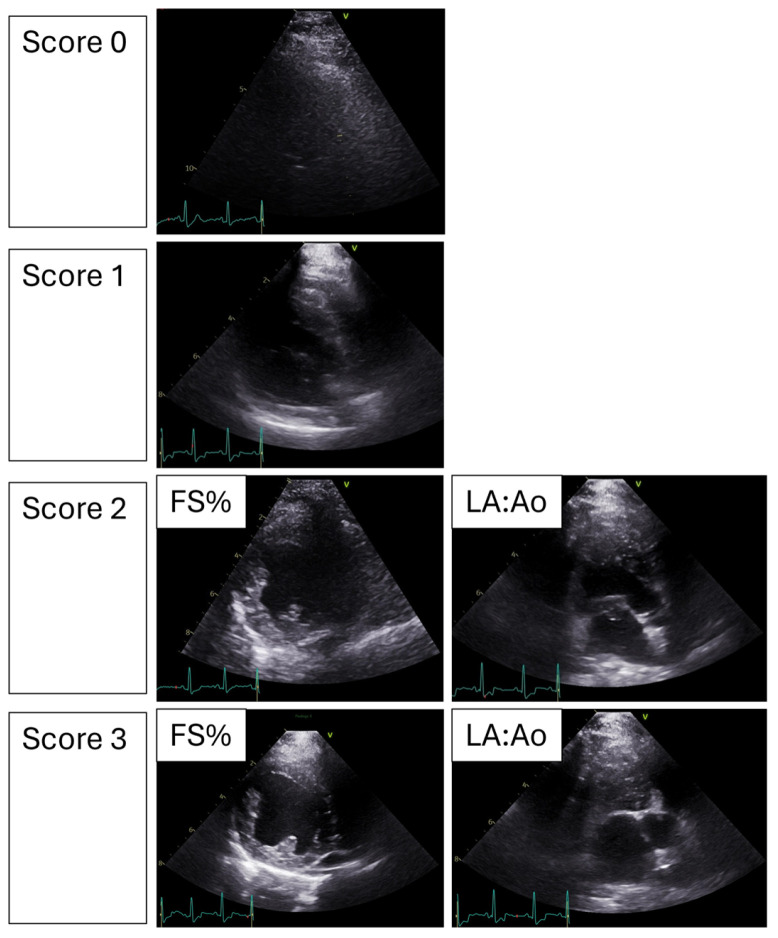
Example images of the quality score system used in the study. FS%—fractional shortening; LA:Ao—left-atrium-to-aorta ratio.

Each cine loop also received an image quality score from the investigator performing the measurements, depicted in Figure 2. The image quality score scale was developed based on a similar human medical study [19]. The image quality score ranged from 0—unable to see the heart; 1—able to see a portion of the heart but unable to measure any meaningful data; 2—able to obtain measurements from some but not all the cardiac cycles; and 3—able to confidently obtain measurements from all cardiac cycles recorded. For example, a score of 0 would be assigned if the heart was not visualizable due to the superimposition of the lungs or poor patient compliance. A score of 1 would be recorded if the heart was visualized but the portions needed for FS% and LA:Ao measurement were obscured, or if the scanning plane was judged to be misaligned relative to the optimal imaging view. A score of 1 would automatically be assigned to the cine loop where only a single measurement of the pair could be obtained, such as being able to measure LIVDs but not LVIDd. A score of 2 would be assigned when measurements were incomplete, meaning they were obtained for some, but not all, recorded heartbeats due to superimposition of the lung, poor patient compliance, or incorrect alignment of the scanning plane. For example, a score of 2 would be assigned if only one set of paired measurements could be obtained from the cine loop. A score of 3 would be a situation where all consecutive heartbeats captured in the cine loop could be measured confidently for all desired variables.

### Statistical Methods

The data sets were visually assessed for normality by using box plots and histograms. For each body position (LT, RT, and RST) and calculated value (FS% and LA:Ao), agreement, clinical assessment, success rate, and image quality were evaluated.

To compare the agreement of the cPOCUS measurements in each position to the echocardiogram measurements, Bland-Altman style plots were created, with the echocardiogram measurement treated as the reference and plotted on the horizontal axis. The median absolute deviation (MAD), the 95% limits of agreement (using mean ± 2 × SD), and the average bias (with standard error and associated *p*-value) are reported, where the *p*-value for the bias was computed using a mixed effect model with a random effect for subject.

Correct clinical assessment was defined as the value for FS% or LA:Ao by cPOCUS in the cine loop having the same clinical assessment, either normal or abnormal, as the echocardiogram. Normal FS% was defined as 25–45%, and normal LA:Ao was defined as ≤1.6. If the cPOCUS was unable to be measured, it was determined that it did not agree with the echocardiogram. The clinical assessment was first described as a proportion of correct assessment compared to the echocardiogram. To assess how the proportion of correct clinical assessment depended on the body position and quality score, generalized estimated equation models were fit with the multiple measures on each individual (across both measurements and investigators) treated as the groups, with exchangeable correlation structure within groups. Models were fit first with only position, and then with a position/quality interaction. For the models with quality, only those with quality scores of 2 or 3 were used, as a quality score of 0 or 1 was defined as being unable to make a measurement and therefore disagreed with the echocardiogram clinical assessment. Proportions and 95% confidence intervals are computed by back-transforming from the logit scale, and pairwise comparisons using Tukey’s honest significant difference adjustment for multiple comparisons.

Success rate was defined as being able to calculate at least one value of either FS% or LA:Ao from a cine loop and was reported as a percentage of the total number of cine loops. Image quality scoring was reported as a percentage of images achieving each score. Intraclass correlation was performed to evaluate for interobserver variability between the different measurers, using a mixed model with a random effect for each individual cine loop. Interobserver agreement was classified as poor (<0.20), fair (0.21 to 0.40), moderate (0.41 to 0.60), good (0.61 to 0.80), and excellent (0.81 to 1.00) [20]. Statistical significance was determined at *p* < 0.05. All computations were performed using R version 4.5.1 (3 June 2025).

## 3. Results

### 3.1. Demographics

Forty dogs met the inclusion criteria and were enrolled in the study. The cPOCUS images for one dog were lost in data transfer; therefore, measurements from 39 dogs were included in the statistical analysis. The mean age was 8.9 years (SD ± 4.1 years). There were 23 female (20 spayed, 3 intact) and 16 male (13 neutered, 3 intact) dogs. The most common breed was Cavalier King Charles Spaniel (*n* = 8), followed by Mixed Breed (*n* = 5), Labrador Retriever (*n* = 4), Chihuahua (*n* = 3), Dachshund (*n* = 2), Doberman Pincher (*n* = 2), Golden Retriever (*n* = 2), Miniature Poodle (*n* = 2), Pomeranian (*n* = 2), and one each of Australian Cattle Dog, Boxer, English Springer Spaniel, French Spaniel, Pug, Rottweiler, Shih Tzu, Vizsla, and Yorkshire Terrier. The median weight was 8.9 kg (range 2.8–46 kg). Five dogs received some form of anxiolytic or sedative prior to the echocardiogram. Three dogs received PO trazodone alone (range 3.55–6.76 mg/kg) prior to the appointment, one dog received IV butorphanol alone (0.3 mg/kg) in the clinic, and one dog received both PO trazodone (4.34 mg/kg) prior to the appointment and IV butorphanol (0.3 mg/kg) in the clinic.

Two dogs had structurally and functionally normal hearts on echocardiogram. Twenty-eight dogs were diagnosed with myxomatous atrioventricular valve disease, four with dilated cardiomyopathy phenotype, five with dysrhythmias, and eight with other primary cardiac diseases. Of the five dogs diagnosed with a dysrhythmia, ventricular premature complexes were identified in three dogs, third-degree AV block with pacing was identified in two dogs, and low-grade AV block was identified in one dog. Other primary cardiac diseases included: pulmonic stenosis (*n* = 2), pulmonary hypertension (*n* = 2), subaortic stenosis (*n* = 1), tricuspid valve dysplasia (*n* = 1), atrioventricular cardiomyopathy (*n* = 1), and intracardiac mass (*n* = 1). Seven dogs had more than one diagnosed cardiac condition. The mean echocardiogram measurement of FS% obtained in 2-D mode was 37.5% (SD ± 11.2%). The mean echocardiogram measurement of LA:Ao was 1.58 (SD ± 0.33). Eighteen dogs had an abnormal FS%, with eight dogs having a FS% between 16% to 25%, and 10 dogs having a FS% between 45% to 56%. Twelve dogs had an abnormal LA:Ao on echocardiogram, ranging from 1.7 to 2.8. The average values for FS% and LA:Ao by echocardiogram and cPOCUS in each body position are described in Table 1.

### 3.2. Agreement Between cPOCUS and Echocardiography Values

**Figure 3:**
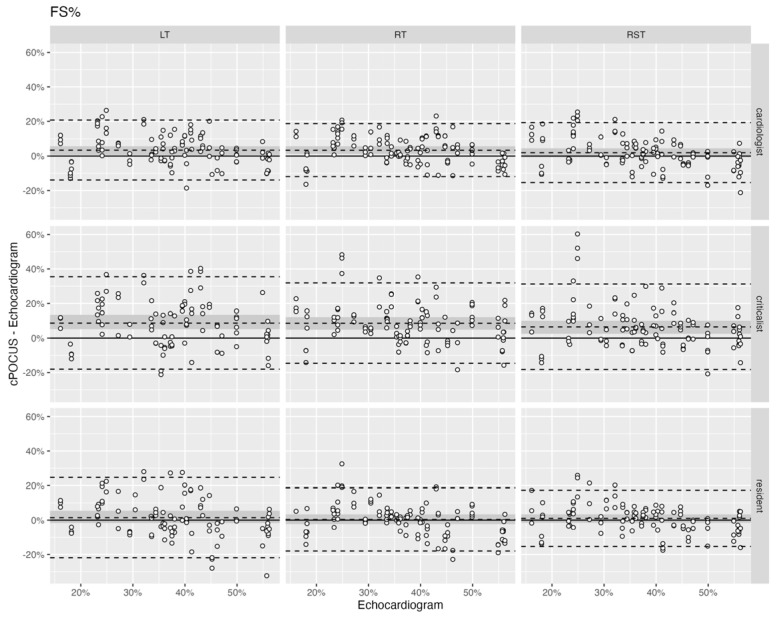
Bland-Altman plot showing the agreement between cardiac point-of-care ultrasound (cPOCUS) and echocardiogram for fractional shortening (FS%) for each of the three body positions. Each row represents an investigator, and each column represents a body position. The echocardiogram value is plotted on the *x*-axis and the cPOCUS comparison on the *y*-axis. Each white dot represents a cPOCUS measurement, as compared to the corresponding echocardiogram measurement. The middle dashed line represents the average bias, and the top and bottom dashed lines represent the 95% limits of agreement. The dark grey area represents the standard error of the average bias. LT—patient in right lateral recumbency with the ultrasound probe on the left hemithorax; RT—patient in left lateral recumbency with the ultrasound probe on the right hemithorax; RST—patient is standing or in sternal recumbency with the ultrasound probe on the right hemithorax.

When comparing FS% obtained in RT, RST, and LT by cPOCUS to the echocardiogram, differences were seen between the different body positions and between cPOCUS and the echocardiogram. Agreement between FS% obtained from cPOCUS images and echocardiogram was evaluated by Bland-Altman style plot analysis, shown in Figure 3. The numerical data is shown in Table A1. The MAD for all investigators ranged from 5% (SD ±8.33) to 11% (SD ±13.7). The narrowest 95% limits of agreement were seen in the RT position for the cardiologist (−12%, 19%) and criticalist (−15%, 32%), and RST for the resident (−15%, 17%). cPOCUS values of FS% had a positive average bias compared to echocardiogram values of FS% for all investigators (0.9% to 9.4%). The average bias was similar in all body positions for the same investigator. The difference in average bias was statistically significant for the cardiologist in the LT (*p* = 0.017) and RT (*p* = 0.003) position, and for the criticalist in all positions (LT: *p* < 0.001; RT: *p* < 0.001; RST: *p* = 0.002) when compared between the investigators. Scatter plot analysis revealed a random scatter with no specific trends and a close relationship to the bias, consistent with no proportional bias.

**Figure 4:**
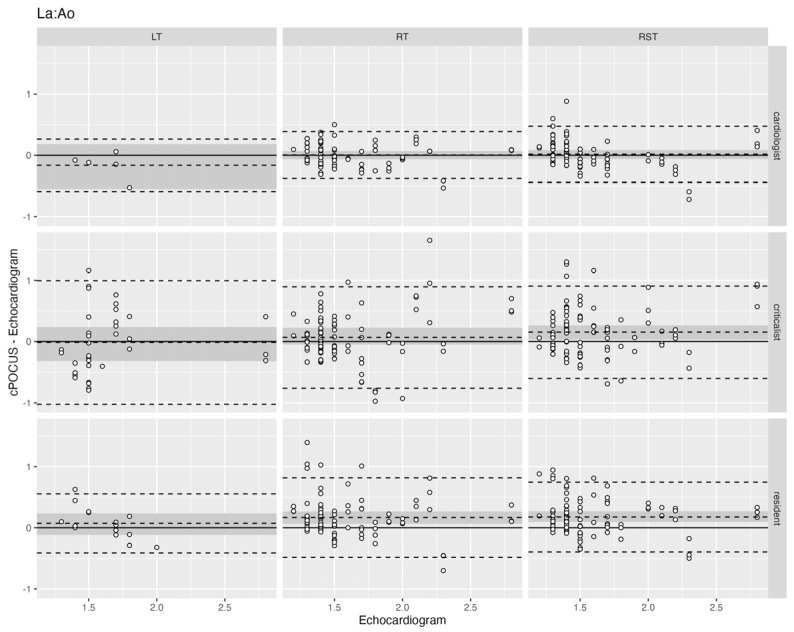
Bland-Altman plot showing the agreement between cardiac point-of-care ultrasound and echocardiogram results for the left-atrium-to-aorta ratio (LA:Ao) for each of the three body positions. Each row represents an investigator, and each column represents a body position. The echocardiogram value is plotted on the *x*-axis and the cPOCUS comparison on the *y*-axis. Each white dot represents a cPOCUS measurement, as compared to the corresponding echocardiogram measurement. The middle dashed line represents the average bias, and the top and bottom dashed lines represent the 95% limits of agreement. The dark grey area represents the standard error of the bias. LT—patient in right lateral recumbency with the ultrasound probe on the left hemithorax; RT—patient in left lateral recumbency with the ultrasound probe on the right hemithorax; RST—patient is standing or in sternal recumbency with the ultrasound probe on the right hemithorax.

When comparing LA:Ao obtained in RT, RST, and LT by cPOCUS to echocardiogram, differences were seen between the different body positions, and between cPOCUS and echocardiogram. Agreement between LA:Ao obtained from cPOCUS images and echocardiogram was evaluated by Bland-Altman plot analysis, shown in Figure 4. The numerical data is shown in Table A2. The MAD for all investigators ranged from 0.11 (SD ±0.25) to 0.40 (SD ±0.51). The narrowest limits of agreement were seen in the RT position for the cardiologist (−0.38, 0.38), in RST for the criticalist (−0.60, 0.91), and in LT for the resident (−0.41, 0.55). cPOCUS values of LA:Ao had a bias within 0.2 of the echocardiogram for all investigators. The average bias was positive in RT and RST for all investigators, and negative in LT for the cardiologist (−0.18, SE ±0.11) and criticalist (−0.04, SE ±0.13). The difference in average bias was statistically significant for the criticalist in the RST position (*p* = 0.015), and the resident in the RT (*p* = 0.002) and RST (*p* < 0.001) positions. Scatter plot analysis showed no proportional bias.

### 3.3. Correct Clinical Assessment by cPOCUS Compared to Echocardiogram

When comparing FS% and LA:Ao obtained by cPOCUS to echocardiogram, the correct clinical assessment of whether the heart was normal or abnormal was more likely in RT and RST positions, compared to the LT position, and was associated with a higher quality score.

For FS%, there was a higher proportion of correct clinical assessments when comparing cPOCUS to echocardiogram measurements in RT and RST compared to LT. For all investigators combined, measurements of FS% lead to a correct clinical assessment an average of 67% of the time in RT, 67% in RST, and 45% in LT. The odds of a correct clinical assessment of FS% in RT were 1.95 times higher than in LT (SE ±0.35; 95% CI: 1.28, 2.97; *p* < 0.001), with an estimated proportion of correct assessment of 0.598 (SE ±0.057; 95% CI: 0.48, 0.70) for RT and 0.433 (SE ±0.051; 95% CI: 0.34, 0.53) for LT. The odds of correct clinical assessment of FS% in RST were 1.78 times higher than with LT (SE ±0.32; 95% CI: 1.16, 2.72; *p* = 0.005), with an estimated proportion of correct assessment of 0.575 (SE ±0.053; 95% CI: 0.471, 0.674) for RST. The differences between RT and RST when assessing correct clinical assessment of FS% were not statistically significant (*p* = 0.82). The clinical assessment for FS% was more often correct when the quality score was 3 compared to 2 in LT, but not RT or RST, with an odds ratio of 1.64 (SE ±1.64; 95% CI: 1.13, 2.40; *p* = 0.01) for LT and estimated odds ratios of 0.76 and 0.95 for RT and RST (*p* = 0.18 and *p* = 0.84, respectively).

For LA:Ao, there was a higher proportion of correct clinical assessments when echocardiogram measurements were compared to cPOCUS in RT and RST compared to LT. For all investigators combined, measurements of LA:Ao had the correct clinical assessment on average: 56% for RST, 45% for RT, and 0% for LT. The odds of a correct clinical assessment of LA:Ao in RT were 8.9 times higher than with LT (SE ±3.41; 95% CI: 3.63, 21.9; *p* < 0.0001), with an estimated proportion of correct assessment of 0.546 (SE ±0.059; 95% CI: 0.43, 0.66) for RT and 0.119 (SE ±0.030; 95% CI: 0.07, 0.19) for LT. The odds of a correct assessment of FS% in RST were 10.2 times higher than with LT (SE ±3.37; 95% CI: 4.77, 21.7; *p* < 0.0001), with an estimated proportion of correct assessment of 0.578 (SE ±0.05; 95% CI: 0.48, 0.67) for RST. The differences between RT and RST when assessing for correct clinical assessment of LA:Ao were not statistically significant (*p* = 0.80). The clinical assessment for LA:Ao was more often correct when there was a higher quality score (a score 3 compared to a score of 2) in RST, but not in RT or LT, with an odds ratio of 0.57 (SE ±0.16; 95% CI: 0.33, 0.98; *p* = 0.042).

### 3.4. Success Rare for Obtaining cPOCUS Images

Obtaining FS% was most frequently achieved in RT and RST positions and slightly less in the LT position for all investigators, as seen in Table 2. The success rate for obtaining FS% was excellent (>81%) for all body positions and similar between investigators. Generally, LA:Ao was more difficult to obtain and was associated with a lower success rate compared to FS%, with the greatest success in the RT and RST positions, and poor ability to obtain measurable images in the LT position. The critical care clinicians obtained more measurements and therefore had higher success rates for obtaining LA:Ao values compared to the cardiologist.

### 3.5. Quality Score of cPOCUS Images Dependent on Body Position

**Figure 5:**
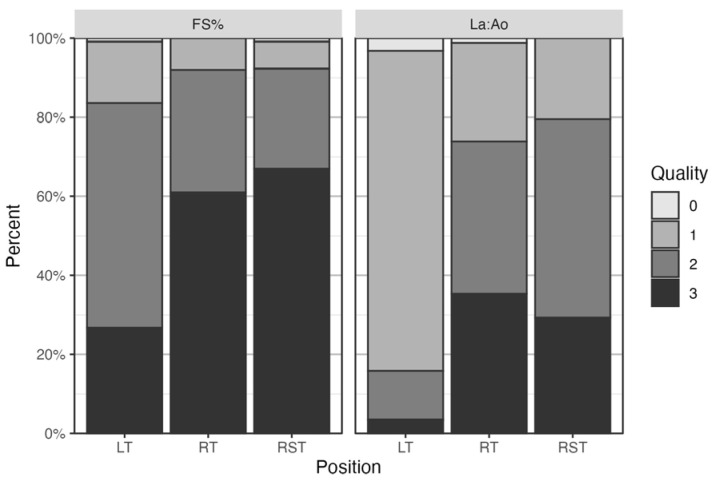
Stacked bar chart showing the average quality scores assigned to the cardiac point-of-care ultrasound cine loops by all investigators combined. Each color represents the quality score (0–3), assigned as a percentage. LT—patient in right lateral recumbency with the ultrasound probe on the left hemithorax; RT—patient in left lateral recumbency with the ultrasound probe on the right hemithorax; RST—patient is standing or in sternal recumbency with the ultrasound probe on the right hemithorax. FS%—fractional shortening; LA:Ao—left-atrium-to-aorta ratio.

Investigators gave the exact same quality score in more than half of the cases, and within 1 point of each other in almost all cases (equal to or above 95% of cases), for both FS% and LA:Ao. On average, the quality score of the FS% images scored comparatively higher than the LA:Ao ones, with FS% cine loops receiving a score of 2 and 3 in over 80% of the cases, as seen in Figure 5. For FS%, all investigators scored the images similarly for quality in each body position, where scores were the highest for RT (a score of 2 or 3 in 92% of cases) and RST (a score of 2 or 3 in 92% of cases). Similarly, for LA:Ao, the highest score was seen in RT (a score of 2 or 3 in 74% of cases) and RST (a score of 2 or 3 in 80% of cases).

### 3.6. Interclass Correlation

Intraclass correlation showed good interobserver agreement between the values of all investigators when compared to each other, as shown in Table 3. However, when each critical care clinician was compared to the cardiologist, poor-to-moderate interobserver agreement was seen for LA:Ao in LT.

## 4. Discussion

Prior investigations have shown the clinical utility of cPOCUS for focused cardiac assessment, with reliable differentiation of normal versus abnormal cardiac structure and function when compared to echocardiography [5,17,21,22,23]. However, patient positioning in these studies has been variably reported, with inconsistent documentation of whether examinations were performed in recumbent or upright positions. Additionally, there is currently no standardization or published consensus regarding optimal body positioning when acquiring a cPOCUS image in dogs.

The results of this study demonstrate that FS% and LA:Ao obtained by cPOCUS performed in different patient body positions were not consistently interchangeable with those obtained by echocardiogram. When high-quality images were obtained, clinical agreement tended to be good. However, when image quality was poor, clinical agreement was more variable, particularly for LA:Ao. Image quality was dependent on the sonographic approach dictated by the patient’s body position. Both FS% and LA:Ao obtained by cPOCUS in the RT and RST positions were associated with a higher quality image and showed a narrow limit of agreement to echocardiogram, compared to the LT position. The findings of this study suggest that of the three positions evaluated in this study, the most reliable way to obtain cPOCUS images is from RT or RST. The use of the LT approach is not advisable.

A positive bias was identified when the cPOCUS results were compared to echocardiography. This is suspected to have been due to obtaining sonographic planes that were less than optimal, for example, capturing slightly oblique images, angling the probe closer to the apex of the heart than ideal echocardiographic standards. For example, when attempting to obtain a transverse image of the aorta, cutting across the aorta at a slight angle would cause it to appear as an oval rather than a circle, and result in a slightly larger measurement. Specifically, more LA:Ao cine-loops scored a 2 or 3 compared to FS%, with obliquity being noted as one of the causes that investigators assigned a lower score.

Measurements could not be performed for all cine loops acquired during the study. The most common reasons were poor patient compliance, the lung and pleural interface obscuring cardiac visualization, only being able to measure one-half of the paired measurements required for the calculation of FS% or LA:Ao, or obtaining the incorrect view—for example, not being able to view the left atrium and aorta in the saved images, which occurred most commonly in the LT view.

Cardiologists typically calculate LA:Ao from the right parasternal short-axis view in 2-D mode from a dog lying in right lateral recumbency [24]. Although both the left atrium and aorta can be visualized in the left apical five-chamber view, this has not previously been described as a way to calculate an LA:Ao value. Nevertheless, this novel approach was included in the study to determine whether LA:Ao measurements could be obtained in situations where the right hemithorax could not be imaged due to patient-related factors. The results of this study support the limited utility of LA:Ao measurements obtained from the left hemithorax.

Roughly half of the dogs enrolled had an abnormal FS%, and over one-third had an abnormal LA:Ao. In this study, we chose a relevant convenience sample of dogs presenting for further cardiac evaluation. Many of the dogs presented for re-examination after having been started on pimobendan, or for evaluation of a dilated cardiomyopathy phenotype, which would increase and decrease the FS% out of the normal reference range, respectively. The most common disease process evaluated for and diagnosed in this study was myxomatous mitral valve disease, which eventually leads to left atrial enlargement. The inclusion of dogs with cardiac pathology could be considered a strength in this study, as it reflects the clinical population in which cPOCUS is performed.

Dysrhythmias observed on lead II ECG were not an exclusion criterion in this study population, as they were present during both echocardiogram and cPOCUS. Where possible, the authors obtained measurements on sinus beats.

Each investigator made a subjective decision about whether measurements could be reasonably obtained based on the degree to which they were able to visualize cardiac structures. The critical care clinicians obtained more measurements and, therefore, had higher success rates for obtaining LA:Ao values, compared to the cardiologist. However, this was accompanied by a higher degree of inaccuracy. The authors speculate that this could be due to the critical care veterinarians measuring images of suboptimal image quality more frequently. It is reasonable to suggest that non-cardiologist clinicians should exercise a greater degree of restraint and caution when determining whether image quality is sufficient to permit reasonably accurate measurement and interpretation of cPOCUS images, especially for LA:Ao. This is particularly important when working under less-than-optimal environments, such as in emergency settings. Regardless of body position, clinicians should prioritize obtaining high-quality images in order to be able to make an appropriate clinical assessment.

This study has several limitations. To acquire the images, a phased array probe and a cardiac ultrasound machine were used to obtain all of the images in this study. In contrast, in most clinical settings, cPOCUS is commonly performed using a micro-convex probe and mobile ultrasound machine [2,6,13]. The quality of the images and accuracy of the measurements obtained in this study may therefore differ from what would be possible to obtain using other sonographic devices.

Utilizing an echocardiographic ultrasound machine also meant that a concurrent lead II ECG was performed. Measurements were performed primarily based on image cues; however, the ECG was used as an additional visual aid, as needed, to identify the specific image frame from which to obtain measurements. For most point-of-care ultrasound machines in the emergency room, measurements are made based on image cues alone. It is possible that measurements acquired from an image that is one or two frames adjacent to the optimal time point may result in a slightly different calculated value for LA:Ao. However, since the intended purpose of cPOCUS is to help veterinarians obtain a general diagnostic impression of cardiac structure and function, rather than precise measurements, these small differences may not be clinically impactful.

An additional limitation was that all of the dogs included in this study were seen on an outpatient and elective basis for a cardiac evaluation. This relatively stable subset of dogs may not be fully representative of the population of dogs undergoing an emergency cPOCUS evaluation due to acute cardiac decompensation. Additionally, whether the interplay of recumbency and decreased intravascular volume might affect the agreement between cPOCUS and echocardiography was not investigated in this study.

## 5. Conclusions

In conclusion, cPOCUS performed in RT and RST can provide estimates of FS% and LA:Ao that are comparable to echocardiography when acquired with good image quality. Imaging of LA:Ao in the LT position is often unsuccessful. Echocardiogram remains the gold standard assessment of the heart structure and function.

## Figures and Tables

**Table 1:** The average measurements and values obtained by echocardiogram and cardiac point-of-care ultrasound. SD—standard deviation; Min—lowest value obtained; Max—highest value obtained; n—total number of values included in study; FS%—fractional shortening; LA:Ao—left-atrium-to-aorta ratio; echo—echocardiogram; RT—patient in left lateral recumbency with the ultrasound probe on the right hemithorax; RST—patient is standing or in sternal recumbency with the ultrasound probe on the right hemithorax; LT—patient in right lateral recumbency with the ultrasound probe on the left hemithorax.

		Mean	SD	Min	Max	n
FS%	Echo	37.5%	11.2	16.1%	56.2%	39
	LT	42.0%	14.4	5.0%	83.3%	291
	RT	41.6%	12.8	1.6%	77.9%	323
	RST	40.5%	12.4	3.7%	85.2%	324
LA:Ao	Echo	1.58	0.33	1.20	2.80	39
	LT	1.63	0.55	0.71	3.21	55
	RT	1.69	0.50	0.83	3.85	257
	RST	1.70	0.46	1.01	3.74	279

**Table 2:** Success rate in obtaining measurements from cardiac point-of-care ultrasound images expressed as a percentage of the total number of cine loops for the specified body position and calculated value. RT—patient in left lateral recumbency with the ultrasound probe on the right hemithorax; RST—patient is standing or in sternal recumbency with the ultrasound probe on the right hemithorax; LT—patient in right lateral recumbency with the ultrasound probe on the left hemithorax; FS%—fractional shortening; LA:Ao—left-atrium-to-aorta ratio.

Investigator	RT		RST		LT	
	FS%	LA:Ao	FS%	LA:Ao	FS%	LA:Ao
Cardiologist	97%	67%	95%	73%	88%	4%
Criticalist	88%	79%	91%	86%	81%	30%
Resident	91%	76%	91%	79%	82%	14%

**Table 3:** Intraclass correlation for the cardiac point-of-care values in different body positions between all investigators and paired investigators. RT—patient in left lateral recumbency with the ultrasound probe on the right hemithorax; RST—patient is standing or in sternal recumbency with the ultrasound probe on the right hemithorax; LT—patient in right lateral recumbency with the ultrasound probe on the left hemithorax; FS%—fractional shortening; LA:Ao—left-atrium-to-aorta ratio.

		All Investigators	Criticalist & Resident	Cardiologist & Resident	Cardiologist & Criticalist
FS%	LT	0.67	0.57	0.73	0.69
	RT	0.64	0.56	0.80	0.59
	RST	0.74	0.64	0.84	0.77
LA:Ao	LT	0.67	0.61	0.20	0.54
	RT	0.73	0.73	0.77	0.69
	RST	0.73	0.81	0.68	0.71

## Data Availability

The raw data supporting the conclusions of this article will be made available by the authors on request.

## Abbreviations

The following abbreviations are used in this manuscript:

cPOCUS	Cardiac point-of-care ultrasound
LA:Ao	Left-atrial-to-aorta ratio
RPSAX	Right parasternal short axis view
FS%	Fractional shortening
ACVIM	American College of Veterinary Internal Medicine
LVIDd	Left ventricular internal diameter at end-diastole
LVIDs	Left ventricular internal diameter at end-systole
LT	Dog in right lateral recumbency with the ultrasound probe on the left hemithorax
RT	Dog in left lateral recumbency with the ultrasound probe on the right hemithorax
RST	Dog standing or in sternal recumbency with the ultrasound probe on the right hemi-thorax
LA	Left atrium diameter
Ao	Aortic diameter
AV	atrioventricular
SD	Standard deviation
MAD	Medial absolute deviation
LOA	Limits of agreement
SE	Standard error
OR	Odds ratio
CI	Confidence interval
PO	per os
IV	intravascular

## Appendix B

**Table A1:** Numerical data obtained from Bland-Altman plot style analysis for cardiac point-of-care ultrasound compared to echocardiogram for fractional shortening. MAD—median absolute deviation; SD—standard deviation of the MAD; LOA—limits of agreement; low—lowest value; high—highest value; delta—absolute difference between the lowest and highest value; SE—standard error for the average bias; RT—patient in left lateral recumbency with the ultrasound probe on the right hemithorax; RST—patient is standing or in sternal recumbency with the ultrasound probe on the right hemithorax; LT—patient in right lateral recumbency with the ultrasound probe on the left hemithorax.

	Investigator	MAD	SD	LOA Low	LOA High	LOA Delta	Bias	SE	*p*-Value
RT	Cardiologist	5.29%	7.81	−11.9%	18.7%	30.6	3.47	1.07	0.003
	Criticalist	8.75%	11.9	−14.6%	31.9%	46.5	8.57	1.80	<0.001
	Resident	5.51%	9.36	−18.0%	18.7%	36.7	0.51	1.38	0.716
RST	Cardiologist	5.24%	8.83	−15.4%	19.3%	34.6	1.88	1.29	0.153
	Criticalist	7.12%	12.6	−18.2%	31.3%	49.5	6.33	1.84	0.002
	Resident	4.82%	8.33	−15.3%	17.3%	32.7	0.90	1.18	0.451
LT	Cardiologist	5.92%	8.84	−13.9%	20.7%	34.6	3.16	1.27	0.018
	Criticalist	11.34%	13.7	−18.0%	35.5%	53.5	9.41	2.05	<0.001
	Resident	6.91%	11.9	−21.9%	24.7%	46.6	1.84	1.78	0.307

**Table A2:** Numerical data obtained from Bland-Altman plot style analysis for cardiac point-of-care ultrasound compared to echocardiogram for the left-atrium-to-aorta ratio. MAD—median absolute deviation; SD—standard deviation of the MAD; LOA—limits of agreement; low—lowest value; high—highest value; delta—absolute difference between the lowest and highest value; SE—standard error for the average bias; RT—patient in left lateral recumbency with the ultrasound probe on the right hemithorax; RST—patient is standing or in sternal recumbency with the ultrasound probe on the right hemithorax; LT—patient in right lateral recumbency with the ultrasound probe on the left hemithorax.

	Investigator	MAD	SD	LOA Low	LOA High	LOA Delta	Bias	SE	*p*-Value
RT	Cardiologist	0.13	0.20	−0.38	0.39	0.77	0.00	0.03	0.952
	Criticalist	0.20	0.42	−0.76	0.90	1.66	0.09	0.07	0.215
	Resident	0.17	0.33	−0.48	0.82	1.30	0.17	0.05	0.002
RST	Cardiologist	0.14	0.23	−0.44	0.48	0.92	0.01	0.04	0.750
	Criticalist	0.21	0.38	−0.60	0.91	1.51	0.15	0.06	0.015
	Resident	0.19	0.29	−0.39	0.75	1.14	0.18	0.04	<0.001
LT	Cardiologist	0.12	0.22	−0.59	0.26	0.86	−0.18	0.11	0.208
	Criticalist	0.40	0.51	−1.02	0.99	2.02	−0.04	0.13	0.746
	Resident	0.11	0.25	−0.41	0.55	0.96	0.06	0.07	0.452

## References

[1] Lisciandro G.R. (2021). Point-of-Care Ultrasound Techniques for the Small Animal Practitioner.

[2] DeFrancesco T.C., Ward J.L. (2021). Focused Canine Cardiac Ultrasound. Vet. Clin. N. Am. Small Anim. Pract..

[3] Kirkpatrick J.N., Panebianco N., Díaz-Gómez J.L., Adhikari S., Bremer M.L., Bronshteyn Y.S., Damewood S., Jankowski M., Johri A., Kaplan J.R. (2024). Recommendations for Cardiac Point-of-Care Ultrasound Nomenclature. J. Am. Soc. Echocardiogr..

[4] Thomas W.P. (1984). Two-dimensional, real-time echocardiography in the dog: Technique and anatomic validation. Vet. Radiol. Ultrasound.

[5] Huh T., Achilles E.J., Massey L.K., Loughran K.A., Larouche-Lebel É., Convey V., McKaba V.F., Crooks A., Kraus M.S., Gelzer A.R. (2024). Utility of focused cardiac ultrasonography training in veterinary students to differentiate stages of subclinical myxomatous mitral valve disease in dogs. J. Vet. Intern. Med..

[6] Burnotte P., Gommeren K., Kennedy C.R., Boysen S.R., Merveille A.-C. (2023). Left atrial measurement in lateral versus sternal recumbency in cats undergoing focused cardiac ultrasound examination. Can. Vet. J..

[7] Foo T.S., Pilton J.L., Hall E.J., Martinez-Taboada F., Makara M. (2018). Effect of body position and time on quantitative computed tomographic measurements of lung volume and attenuation in healthy anesthetized cats. Am. J. Vet. Res..

[8] Ahlberg N.E., Hoppe F., Kelter U., Svensson L. (1985). A computed tomographic study of volume and x-ray attenuation of the lungs of beagles in various body positions. Vet. Radiol. Ultrasound.

[9] Greco A., Meomartino L., Raiano V., Fatone G., Brunetti A. (2008). Effect of left vs. right recumbency on the vertebral heart score in normal dogs. Vet. Radiol. Ultrasound.

[10] Ruehl W.W., Thrall D.E. (1981). The effect of dorsal versus ventral recumbency on the radiographic appearance of the canine thorax. Vet. Radiol. Ultrasound.

[11] Ashdown R.R., Done S. (2009). Color Atlas of Veterinary Anatomy Volume 3: The Dog & Cat.

[12] Keene B.W., Atkins C.E., Bonagura J.D., Fox P.R., Häggström J., Fuentes V.L., Oyama M.A., Rush J.E., Stepien R., Uechi M. (2019). ACVIM consensus guidelines for the diagnosis and treatment of myxomatous mitral valve disease in dogs. J. Vet. Intern. Med..

[13] Janson C.O., Hezzell M.J., Oyama M.A., Harries B., Drobatz K.J., Reineke E.L. (2020). Focused cardiac ultrasound and point-of-care NT-proBNP assay in the emergency room for differentiation of cardiac and noncardiac causes of respiratory distress in cats. J. Vet. Emerg. Crit. Care.

[14] Hezzell M.J., Ostroski C., Oyama M.A., Harries B., Drobatz K.J., Reineke E.L. (2020). Investigation of focused cardiac ultrasound in the emergency room for differentiation of respiratory and cardiac causes of respiratory distress in dogs. J. Vet. Emerg. Crit. Care.

[15] Ward J.L., Lisciandro G.R., Ware W.A., Viall A.K., Aona B.D., Kurtz K.A., Reina-Doreste Y., DeFrancesco T.C. (2018). Evaluation of point-of-care thoracic ultrasound and NT-proBNP for the diagnosis of congestive heart failure in cats with respiratory distress. J. Vet. Intern. Med..

[16] Visser L.C., Ciccozzi M.M., Sintov D.J., Sharpe A.N. (2019). Echocardiographic quantitation of left heart size and function in 122 healthy dogs: A prospective study proposing reference intervals and assessing repeatability. J. Vet. Intern. Med..

[17] Martin-Flores M., Desrochers A.L., Rishniw M., Araos J. (2025). Visual (eyeball) estimation by observers with varying echocardiographic experience reliably identifies severe but not moderate decreases of the fractional shortening in dogs. Am. J. Vet. Res..

[18] Hansson K., Häggström J., Kvart C., Lord P. (2002). Left atrial to aortic root indices using two-dimensional and M-mode echocardiography in cavalier King Charles spaniels with and without left atrial enlargement. Vet. Radiol. Ultrasound.

[19] Millington S.J., Arntfield R.T., Hewak M., Hamstra S.J., Beaulieu Y., Hibbert B., Koenig S., Kory P., Mayo P., Schoenherr J.R. (2016). The Rapid Assessment of Competency in Echocardiography Scale: Validation of a Tool for Point-of-Care Ultrasound. J. Ultrasound Med..

[20] Altman D.G. (1990). Practical Statistics for Medical Research.

[21] Dickson D., Harris J., Chang C., Patteson M., Hezzell M.J. (2022). Validation of a focused echocardiographic training program in first opinion practice. J. Vet. Intern. Med..

[22] Lyssens A., Lekane M., Gommeren K., Merveille A.-C. (2022). Focused Cardiac Ultrasound to Detect Pre-capillary Pulmonary Hypertension. Front. Vet. Sci..

[23] Tse Y.C., Rush J.E., Cunningham S.M., Bulmer B.J., Freeman L.M., Rozanski E.A. (2013). Evaluation of a training course in focused echocardiography for noncardiology house officers. J. Vet. Emerg. Crit. Care.

[24] Kuo M.Y.W., Häggström J., Gordon S.G., Höglund K., Côté E., Lu T.-L., Dirven M., Rishniw M., Hung Y.-W., Ljungvall I. (2024). Veterinary echocardiographers’ preferences for left atrial size assessment in dogs: The BENEFIT project. J. Vet. Cardiol..

